# Nephrectomy for Sarcoma, with Report of Case and Exhibition of Specimen*Read before Georgia Medical Association, Atlanta, Ga., April, 1894.

**Published:** 1894-05

**Authors:** J. B. S. Holmes

**Affiliations:** Atlanta, Ga.; President of the Tri-State Medical Association; Vice-President of the Southern Surgical and Gynecological Association; Ex-President of the Georgia State Medical Association; Fellow of the American Association of Obstetricians and Gynecologists, and Member of the American Medical Association


### NEPHRECTOMY FOR SARCOMA, WITH REPORT OF CASE AND EXHIBITION OF SPECIMEN. *

By J. B. S. HOLMES, MD., ATLANTA, GA.

President of the Tri-State Medical Association ; Vice-President of the Southern Surgical and Gynecological Association ; Ex-President of the Georgia State Medical Association ; Fellow of the American Association of Obstetricians and Gynecologists, and Member of the American Medical Association.

Mary N., aged 13 months, family history free from cancer, tuberculosis, or syphilis, rather small for her age, but in good health up to early in January of the present year, when she became irritable with considerable digestive disturbance, accompanied with loose bowels. Her mother at this time, in examining her abdomen, detected a small growth about the size of a hen's egg in the right lumbar region. She consulted her family physician, who diagnosed the case as enlargement of the liver. The child was continued under his treatment until the latter part of March, when she was taken to my friends, Dr. R. M. Harbin, of Calhoun, and W. C. Nixon, of Nannie, Ga. Both gentlemen kindly referred the case to me. The child was brought to my office on the 6th inst. After a thorough examination my diagnosis was given as sarcoma of the right kidney. Dr. Garlington subsequently saw the case with me and concurred in this opinion. After explaining the nature of the trouble to the parents, stating that it would prove fatal in a short time and suggesting no hope except from surgical procedure, of course explaining to them the dangers attendant upon it and giving them little encouragement from this source, both the parents not only readily assented to an operation but urged it. The child was brought to me again on the 9th inst. for operation. A dose of oil was immediately given with instructions to the mother to allow nothing in the stomach after six o'clock on the following morning. The next morning, after a bath all over, a nephrectomy was done. After thoroughly cleansing the abdomen, an incision was made beginning near the lower border of the eighth rib and extending downward on the outer side of the linea semilunaris to near

*Read before Georgia Medical Association, Atlanta, Ga., April, 1894.

Poupart's ligament. As the tumor was very large, pushing the peritoneum far over to the left, I had hoped to do an extraperitoneal operation, but upon making my incision and reaching the capsule I found it so firmly adherent to the peritoneum as to be unable to separate it without risk of doing serious damage, and perhaps badly lacerating this membrane. Again, on account of the immense size of the growth I was unable to enucleate it without going into the abdominal cavity. I was forced also to make a transverse incision extending from about the middle of the longitudinal one three inches in the direction of the spine. After considerable difficulty the capsule was detached from the peritoneun and the tumor turned out. It was my purpose to have ligated the artery and vein together and the ureter separately, but as considerable time had been consumed in breaking up the adhesions, I ligated the ureter and vessels *en masse* with a silk ligature. The peritoneum was closed without flushing by a running suture of fine silk. I did not include the peritoneum in the stiches through the skin, muscle and fascia, from the fact that I had to go through so much muscular tissue and fascia with the resulting uneven retraction of the fascia that I was afraid I could not get perfect coaptation of like tissue by passing my sutures from the skin to the peritoneum, including all intervening substance.

A drainage tube of rubber was placed in the lower angle of the wound, extending from the cut vessels to two inches beyond the skin. This tube was allowed to remain forty-eight hours, when it was removed, the opening closing promptly without suture. Before closing the wound the tissues were thoroughly cleansed with sterilized water. After bringing the parts together iodoform was sprinkled freely over the surface, four thicknesses of iodoform gauze were laid over the abdomen, bichloride cotton placed upon this and two adhesive strips two inches wide placed over this, extending two-thirds around the body.

Squibb's Chloroform was used as the anesthetic. The patient was put to bed in good condition, having lost not more than an ounce of blood, perhaps not so much. At the end of twelve hours her temperature had reached 100°. The following day at 3 p. m., twenty-eight hours after the operation, her temperature had reached

$102\frac{1}{2}°$; considerable tympanitic distension of the abdomen, but no gastric disturbance had shown itself. I gave her one grain each of calomel and soda, with instructions that a half grain of each be given every four hours till three grains had been taken. It was not necessary, however, to repeat the dose as the first acted within three hours, and acted three times afterwards within twelve hours, she passing six immense lumbricoid worms in this time. The following day her temperature was normal and continued so until the fourth day, when there was a slight elevation, reaching 100°. I examined the dressings and found some redness at one stitch which had been drawn too tight. This was removed and a little pus escaped, when the temperature went down and in six hours was normal, continuing so up to the time I left her on Tuesday afternoon, the beginning of the eighth day. Half of the stitches were removed on the sixth day and the balance the day following. With the exception of one stitch hole abscess, the incision healed entirely without suppuration. She has had no nourishment from the beginning except her mother's milk, which I have allowed her to have freely after the first twenty-four hours. She has also after that time had water *ad libitum.* No opiate has been given. When I last saw her, Tuesday afternoon, she was playing with her toys resting upon an easy pillow in her little carriage, and I feel perfectly assured as to the result of the operation.

The tumor, with the kidney, which I have the pleasure to present to the Association, weighed three pounds and two ounces. As you will see, the kidney is not incapsulated by it, but rests almost as a flattened shell upon its surface.

I have not had my diagnosis verified by microscopic examination, which I will have done later. The case, on account of the youth of the patient, is one of considerable interest. I might add that when the abdomen was opened, before removing the tumor, I passed my hand to see if the left kidney existed, and I found that it did, and as far as I could tell from touch it was sound. She has secreted an abundance of urine since the operation.

I was assisted in the operation by Drs. Garlington, Johnson and Wynn.

NOTE—Since this paper was read the patient has been discharged, cured.

